# Non-Specific Low Back Pain: An Inductive Exploratory Analysis through Factor Analysis and Deep Learning for Better Clustering

**DOI:** 10.3390/brainsci13060946

**Published:** 2023-06-13

**Authors:** Lucien Robinault, Imran Khan Niazi, Nitika Kumari, Imran Amjad, Vincent Menard, Heidi Haavik

**Affiliations:** 1 Centre for Chiropractic Research, New Zealand College of Chiropractic, Auckland 1060, New Zealand; lucien.robinault@nzchiro.co.nz (L.R.); imran.niazi@nzchiro.co.nz (I.K.N.); nitika.kumari@nzchiro.co.nz (N.K.); imran.amjad@nzchiro.co.nz (I.A.); 2 Faculty of Health and Environmental Sciences, Health and Rehabilitation Research Institute, AUT University, Auckland 1010, New Zealand; 3 Department of Health Science and Technology, Aalborg University, 9220 Aalborg, Denmark; 4 Faculty of Rehabilitation and Allied Health Sciences and Department of Biomedical Engineering, Riphah International University, Islamabad 46000, Pakistan; 5 M2S Laboratory, ENS Rennes, University of Rennes 2, 35065 Rennes, France; menardvincent18@hotmail.fr

**Keywords:** low back pain, motion capture, high-density electromyography, factor analysis, deep learning

## Abstract

Non-specific low back pain (NSLBP) is a significant and pervasive public health issue in contemporary society. Despite the widespread prevalence of NSLBP, our understanding of its underlying causes, as well as our capacity to provide effective treatments, remains limited due to the high diversity in the population that does not respond to generic treatments. Clustering the NSLBP population based on shared characteristics offers a potential solution for developing personalized interventions. However, the complexity of NSLBP and the reliance on subjective categorical data in previous attempts present challenges in achieving reliable and clinically meaningful clusters. This study aims to explore the influence and importance of objective, continuous variables related to NSLBP and how to use these variables effectively to facilitate the clustering of NSLBP patients into meaningful subgroups. Data were acquired from 46 subjects who performed six simple movement tasks (back extension, back flexion, lateral trunk flexion right, lateral trunk flexion left, trunk rotation right, and trunk rotation left) at two different speeds (maximum and preferred). High-density electromyography (HD EMG) data from the lower back region were acquired, jointly with motion capture data, using passive reflective markers on the subject’s body and clusters of markers on the subject’s spine. An exploratory analysis was conducted using a deep neural network and factor analysis. Based on selected variables, various models were trained to classify individuals as healthy or having NSLBP in order to assess the importance of different variables. The models were trained using different subsets of data, including all variables, only anthropometric data (e.g., age, BMI, height, weight, and sex), only biomechanical data (e.g., shoulder and lower back movement), only neuromuscular data (e.g., HD EMG activity), or only balance-related data. The models achieved high accuracy in categorizing individuals as healthy or having NSLBP (full model: 93.30%, anthropometric model: 94.40%, biomechanical model: 84.47%, neuromuscular model: 88.07%, and balance model: 74.73%). Factor analysis revealed that individuals with NSLBP exhibited different movement patterns to healthy individuals, characterized by slower and more rigid movements. Anthropometric variables (age, sex, and BMI) were significantly correlated with NSLBP components. In conclusion, different data types, such as body measurements, movement patterns, and neuromuscular activity, can provide valuable information for identifying individuals with NSLBP. To gain a comprehensive understanding of NSLBP, it is crucial to investigate the main domains influencing its prognosis as a cohesive unit rather than studying them in isolation. Simplifying the conditions for acquiring dynamic data is recommended to reduce data complexity, and using back flexion and trunk rotation as effective options should be further explored.

## 1. Introduction

Non-specific low back pain (NSLBP) is characterized by pain for more than one day located between the lower rib margins and the buttock creases. The pain does not have a definite cause and cannot be traced to a specific event or affliction [1]. It can be acute, or chronic, if it lasts more than 3 months continuously, and it might or might not limit the person’s usual activities or change their daily routine [2]. NSLBP is a complex symptom that is known to encompass multiple aspects of the patient’s profile. Those aspects can be classified into five categories: biophysical, social, psychological, and genetic factors, and comorbidities [3]. LBP is a long-lasting and, frequently, recurring symptom: 76% of people complaining of LBP report previous episodes [4]. LBP is extremely prevalent in the population worldwide, with a one-year point prevalence of 38% [5] and a global point prevalence of 7.3%, which translates to 540 million people affected at any time in the world [3]. Out of all the LBP diagnoses, between 90% and 99% are deemed to be non-specific [6,7,8,9,10].

According to global estimates, the point prevalence of low back pain (including acute, subacute, and chronic) was 7.83% in 2017, with approximately 577 million people affected at any given time. In 2015, low back pain was responsible for 60.1 million disability-adjusted life years [11]. The clinical course of LBP tends to be favorable, with more than 76% of individuals recovering within three months of an episode [4]. However, approximately 70% of individuals experience a recurrence of LBP within 12 months following recovery [12]. The progression to chronicity is associated with significant disability and costs for society [13]. LBP ranks the highest in terms of disability and sixth in terms of overall burden, as measured by disability-adjusted life years (DALYs) [14]. In fact, LBP contributes to a greater number of individuals leaving the labor force compared to several other common conditions combined, such as diabetes, hypertension, neoplasms, asthma, and heart and respiratory diseases [15].

The Clinical Practice Guidelines for the Management of Acute and Chronic Low Back Pain provide a range of interventions for the management of low back pain, including patient education, exercise therapy, manual therapy, and psychological interventions [16]. A recent systematic review and meta-analysis investigating the most effective treatments for acute and subacute mechanical non-specific low back pain found that cognitive behavioral therapy (CBT) was the most effective treatment, followed by spinal manipulation, massage, and acupuncture [17].

NSLBP is a significant public health problem worldwide [14]. It is the leading cause of disability and costs governments and insurance providers billions of USD in treatments and workers’ compensation costs every year [14]. The problem is present in developed countries as well as developing ones, but presents different issues in both: in developed countries, the impact and burden of NSLBP is placed on the healthcare and social system first. In contrast, in developing countries, the burden is predominantly placed on the people impacted by LBP and their support systems [3]. Currently, NSLBP is an epidemic that is both costly and socially debilitating [14], and despite researchers and public health officials’ best efforts over several decades, the problem is becoming worse [14].

The lack of knowledge on and treatment for NSLBP does not come from a lack of research and clinical effort, but from the complexity of the task at hand. The major problem of NSLBP is that it affects a very large and heterogeneous population. This makes it difficult to study the symptom, and to design treatments or rehabilitation protocols that are effective for everyone affected [18]. Today, practitioners have to rely mostly on a “one size fits all” approach, with relatively ineffective generic rehabilitation protocols [1].

One emerging solution to overcoming this issue is to cluster the NSLBP population into more homogeneous subgroups in order to facilitate the development of more targeted care, which should be more effective than the currently available tools [3,18,19,20]. However, so far, the NSLBP population has not been able to be subdivided in reliable and clinically meaningful clusters [1].

If clustering of the NSLBP population was achieved, the problem’s complexity would be reduced due to the lower diversity among clusters. Those clusters would then be easier to study due to their increased homogeneity, which would hopefully lead to more applicable knowledge in the clinical field, notably in the design of targeted rehabilitation protocols. Indeed, instead of studying an extremely heterogeneous population, researchers and clinicians would be facing a homogeneous sub-population. However, at present, the research community has yet to find valuable solutions to the clustering task [1,3,21]. In the past, few of the classification models designed in research were used in a clinical setup, or even used in the field. Moreover, the few clinically usable classification models have a very variable usage rate: between 7% and 70% [22,23,24,25,26,27,28].

Today, most attempts to classify NSLBP rely on the use of categorical data. Most of the current classification models that work do so using questionnaire data, or variables in general, that are categorical at the time of acquisition [21,29]. The categorization, as well as answering those categorical variables, are subjective acts. This arbitrary aspect might limit the extrapolation of those clusters, found through those categorical models, into actual useful groups in a clinical setting. The work of the Quebec Task Force is a good example of this [30]. Being able to design classification models based on objective data would provide the classification models with a more solid foundation. Some experiments have attempted to circumvent the difficulties of designing classification models using strict continuous data by categorizing those continuous variables, some with appreciable results [31]. While this is a better solution than relying on a subjective assessment from the subject, it still poses the issue of subjectivity: the categorization relies on an educated guess by the operator, and is therefore tied to their judgment, appreciation, and subjectivity.

There is a need to better understand continuous variables in relation to NSLBP, in different conditions, to facilitate the development of clinically relevant clusters among the NSLBP population. An important step in designing rehabilitation protocols tailored to NSLBP patients’ needs is providing clustering models closer to the clinical reality. This is a goal that, we think, can be reached via the use of the more objective measurement of recorded continuous variables. Therefore, this study aimed to provide exploratory work to better understand the influence and importance of variables related to NSLBP and how to use those variables to facilitate clustering.

## 2. Materials and Methods

### 2.1. Population

At first, 53 subjects were recruited and divided into 2 groups: Healthy, 17 subjects; and NSLBP, 36 subjects. A total of 7 subjects were excluded from the data set post-acquisition due to issues during the data collection that compromised data quality, bringing the total number of subjects to 46:14 Healthy and 32 NSLBP. The population characteristics are displayed in Table 1.

The subjects were recruited via advertising within the New Zealand College of Chiropractic community. The participants interested in the study were to contact the person in charge of recruitment via e-mail or telephone. They were then invited for a quick interview to assess if they fit the inclusion criteria of our study. During the interview, the recruiter mentioned that a 20 NZD petrol voucher was offered to the participant at the end of the acquisition session for their time and effort if they partook in the study. Participants included students, staff, faculty, and previous patients of the college’s chiropractic center, as well as family, friends, and acquaintances of the New Zealand College of Chiropractic community. The experimental protocol was advertised by inviting volunteers to contact our team in order to participate in a scientific study, whether they have experienced lower back pain and either received treatment or not (the NSLBP group), or they have not experienced back pain (the Healthy group).

The population inclusion/exclusion criteria were as follows:

- English speaking;
- Age between 18 and 50;
- In case of LBP, no identifiable cause for it;
- Not suffering from a current lower limb disorder/dysfunction;
- No recent history of inner ear infection with associated balance or coordination problems;
- No history of cerebral trauma with unresolved sensorimotor symptoms;
- No recent history of vestibular disorder;
- No previous spinal surgery;
- No involvement in specific balance or stabilization training in the past 6 months;
- Not currently on pain medication;
- Below 3/10 on the pain scale on the day of data collection [32].

NSLBP was defined as pain located between the lower rib margins and the buttock crease [2] for which the patho-anatomical cause was not determined [1]. The LBP must have been present at least 3 months in the last year to be counted as chronic [33].

### 2.2. Location

Data acquisition was conducted at the Auckland University of Technology North Campus’s motion capture laboratory, Auckland, New Zealand. Participants were not blinded to group allocation. Outcome assessors and data analysts remained blinded to group allocation throughout the data collection and data processing steps.

### 2.3. Protocol

This study was an inductive exploratory analysis, which the Health and Disability Ethics Committees approved on 6 November 2019 (Approval: 19/CEN/187).

**Figure 1:**
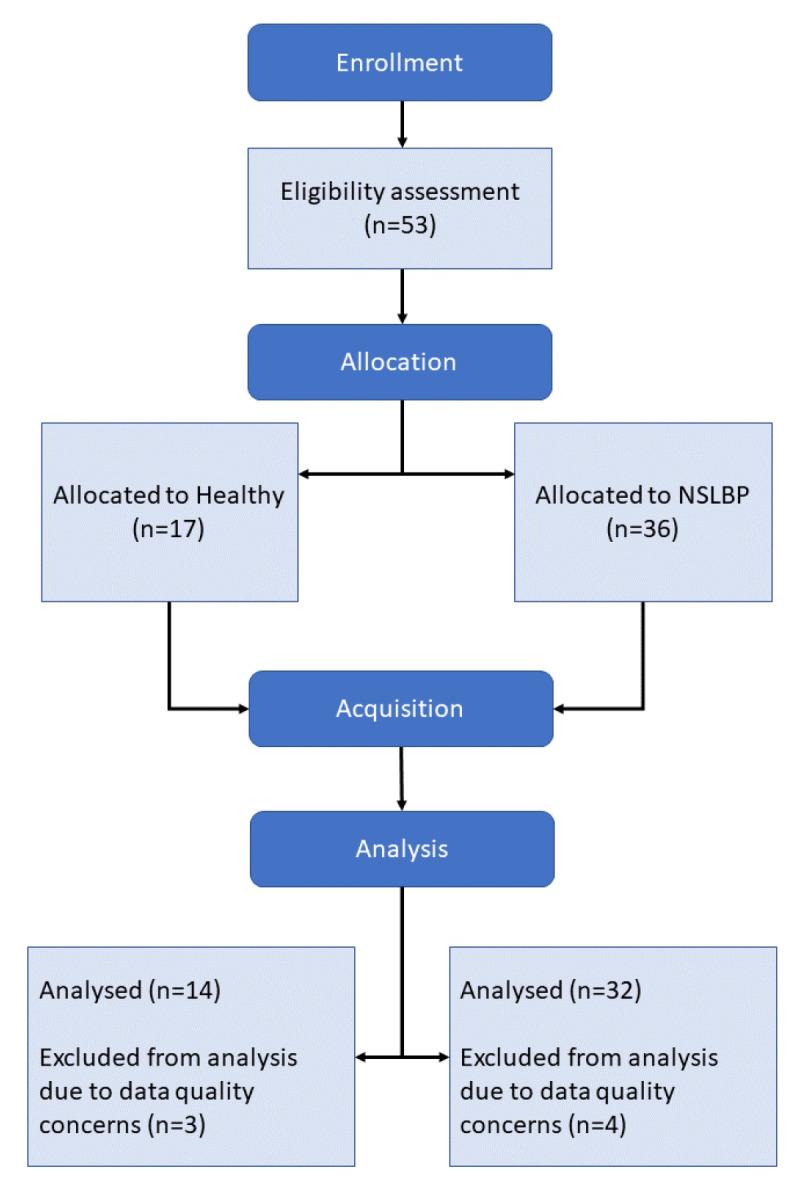
Experimental setup.

The experiment, as described in the flow chart in Figure 1, was conducted as follows, and took around 2.5 h to run for each participant:

- Consent collection;
- Subject preparation;
- Static postural recording;
- Movement execution at preferred speed;
- Static postural recording;
- Movement execution at maximal speed;
- Static postural recording;
- Subject release.

#### 2.3.1. Subject Preparation

Once subjects arrived in the lab, they were given an explanation of their rights, the experimental procedures, and the safety rules of the lab. A written consent form and a participant information sheet, detailing the aforementioned information in writing, were provided to the participants for review and signature before starting the experiment. The participants then provided informed consent if they accepted to participate in the study.

**Figure 2:**
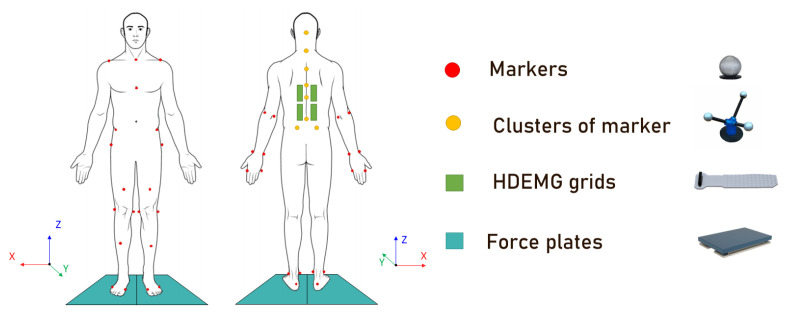
Experimental setup.

Spherical reflective surface markers (7 mm) and clusters of markers (6 mm), custom made by Optitrack (NaturalPoint, Inc. DBA Optitrack, Corvallis, OR, USA), were placed on anatomical landmarks according to the International society of Biomechanics’s recommendation [34,35] to make up a model of 38 markers on the body, and 8 clusters of markers were placed on the spine as shown in Figure 2. The cluster of markers allowed for the creation of multiple segments of the spine. The full set divided the spine into 5 segments: Hips–L5, L5–T12, T12–T6, T6–T3, and T3–C7. These positions for the clusters were chosen as they allow for a fine representation of spinal kinematics with a minimal number of clusters of markers [36,37]. The clusters of markers were homemade using the following structure: a base made of a thin acrylic square, to which a neodymium magnet and 3 aluminum sticks were bound with epoxy. At the end of the sticks, 6 mm diameter passive reflective markers were attached. The structure made of the sticks was reinforced with tape. We made sure that each pair made by combining cluster markers did not end up forming a parallel axis. Magnets were used for the cluster fixation on the spine of the subject. The magnets were placed on the respective spinous processes via the use of double-sided tape (Vicon Motion Systems Ltd., Oxford, UK).

For high-density electromyography (HD EMG), the area of skin where the electrodes were to be placed was prepared by gentle abrasion using abrasive paste (Everi, Spes Medica, Genova, Italy); then, the area was cleaned with alcohol wipes, following recommendations [38]. To record HD EMG signals, we used the HD EMG Quattrocento amplifier (OT Bioelettronica, Turin, Italy) alongside a semi-disposable adhesive electrodes matrix from the same company, model GR08MM1305 with a 5-column, 13-row architecture, for 63 electrodes with a diameter of 1 mm and inter-electrode distance of 8 mm. The GR08MM1305 patches of electrodes were prepared by first applying an adhesive foam on them, which was filled with a conductive and adhesive paste, AC Cream (Spes Medica S.r.l., Genova, Italy). The electrode grids were placed on either side of the lower back, always starting from the lower pair, from L5 to L2, depending on the subject morphology, to the upper pair, from L1 to T8, again depending on the subject morphology [39,40,41]. The grids were placed around 2 cm away from the spine so as to cover the back extensors and paraspinal muscles [42]. Figure 2 displays the grid of the electrode placement.

Once the patches of electrodes were placed, Pretaping Adhesive Spray Grip (D3, London, England) was sprayed on the spine area to increase the adhesive power of the X6.0 D3 tape (D3, London, England) used to secure the magnets onto the spine of the subject. The same X6.0 tape was applied around the waist of the subject to secure the patches of electrodes to the subject’s body in order to minimize their displacement during the subject’s movements. The reference electrodes were placed on the crest of the spine of the scapula. The subject’s reference for the amplifier was fixed on their ankle.

#### 2.3.2. Data Acquisition

The HD EMG signals were acquired using the software OTBiolab+ (version 1.5.7.3, OT Bioelettronica, Turin, Italy). The settings used were:

- Bandpass filter: 10–500 Hz;
- Sampling frequency: 2048 Hz;
- Gain: 2000.

A D64 HD EMG adaptor (OT Bioelettronica, Turin, Italy) was used to connect the patches of electrodes to the amplifier. All the parameters were manipulated from the interface of the OT Bioelettronica software.

Our motion capture (MOCAP) system was a Qualisys system (Qualisys AB, Göteborg, Sweden, version 2021.2, build 6940) with a 9 Oqus 500+ series camera and a sampling rate of 120 Hz. Two Amti force platforms, S464508 series (Watertown, MA, USA), were connected to the Qualisys system via an analog interface USB-2533 (Measurement Computing Corporation DAQ) with an acquisition rate of 1000 Hz.

Once the subject was prepared, they were set in the center of the acquisition volume of the MOCAP system, each foot on a separate force plate. The subject was instructed to take a natural foot stance. Aside from the fact that each foot had to remain on its respective force plate, the foot stance was not controlled [43]. Indeed, foot stance is subject to be different between healthy and LBP subjects [44].

Once the subject was in position, they were given instructions on how to perform each movement:

- Static postural recording, 90 s eyes open and 3 × 90 s eyes closed;
- Movement tasks, 10 repetitions each, eyes closed, at preferred velocity and maximal velocity:
  − Back extension;
  − Back flexion;
  − Lateral trunk flexion, left and right;
  − Trunk rotation, left and right.

**Figure 3:**
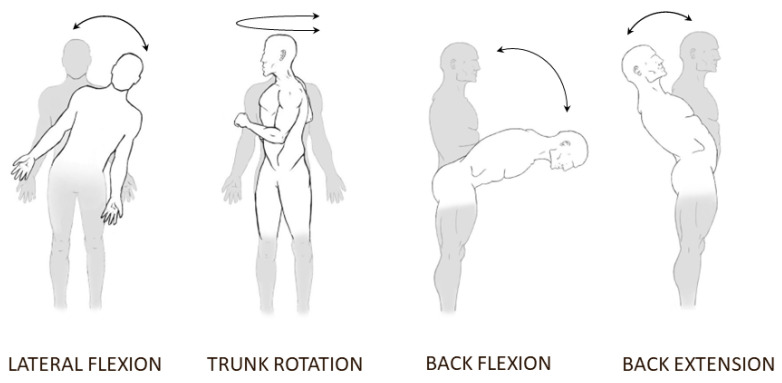
Experimental setup.

The static postural task consisted of the subject standing upright, straight, and relaxed, and to not move for the duration of the task. Two static postural recordings were performed first, one with eyes closed and one with eyes open; then, another recording with eyes closed after the movements at preferred speed had been performed; and a last recording with eyes closed after the movements at maximal speed had been performed. The movement tasks were performed in a random order, different between preferred and maximal speed. All conditions, except for the first static postural recordings, were executed with eyes closed in order to maximize the difference between the two groups [45,46]. Flexion, extension, and abduction of the lower limb joints were monitored during movement tasks. Repetition was invalidated if it occurred. The different movement tasks are displayed in Figure 3.

The preferred velocity was defined as:

The speed at which the subject would perform the movement in day-to-day life.

The maximal velocity was defined as:

The maximal speed at which the subject can perform the movement safely.

For the lateral bending task, participants were asked to bend their trunk laterally from an upright posture to their maximal lateral flexion, without engaging the hip joint or bending their knees.

For the trunk rotation task, participants were asked to rotate their trunks from the starting position, without engaging the hip joint or bending their knees. For the back extension task, the participants were asked to extend their back backward, without bending their knees.

For the forward bending task, participants were asked to bend their trunk without moving their hip joints or bending their knees. The forward bending task had a slightly different execution than the other movements when performed at the preferred speed, in order to study the flexion relaxation response [47,48]. To achieve this, the subject performed the actual trunk flexion movement, from a standing position to the maximum amplitude, in 3 s. Once the maximum amplitude was reached, the subject stayed still in that position for 3 s. The subjects then returned to their starting standing position, again in 3 s.

Once explanations were given and understood, the protocol itself started. The subject was asked to perform the movements, 10 consecutive repetitions each, first at the preferred speed and then at maximum speed. The repetitions were made to be as independent as possible by waiting until the participant stabilized at the end of each repetition before engaging in a new repetition. This was controlled by the MOCAP operator via the force plate readings. The movements were performed in a randomized order. The randomized order was different for the preferred and maximum speed movements.

### 2.4. Materials and Data Analysis

We used a deep neural network (DNN) and factor analysis (FA) to conduct our exploratory analysis. The DNN was used to classify between healthy and NSLBP subjects. Different models were assessed, each using a different set of variables. The information yielded by each type of variable was assessed through the classification accuracy of the model using them. Below is a summary of the models assessed:

- Full: Null model to assess the capacity of our data to classify the two populations;
- Anthropometric: Model to assess the information yielded by the anthropometric data;
- Balance: Model to assess the information yielded by the stabilometric and balance data from the force plates;
- Biomechanical: Model to assess the information yielded by the biomechanical data from the motion capture;
- Neuromuscular: Model to assess the information yielded by the neuromuscular data from the HD EMG.

The models were trimmed in order to gain a finer understanding of different subdomains’ importance by only focusing on the variables of the respective subdomains. The variables used by each model, full and trimmed, are detailed below in the Variables Used and DNN sections. The DNN models are movement agnostic and encompass the data from all of the movements performed.

In addition to this, an exploratory FA was performed. The goal was to assess which variable correlated to NSLBP, with which intensity, and in which “direction”, positively or negatively for each movement, in order to obtain a finer understanding of the relationship of the different variables and factors associated with NSLBP.

#### 2.4.1. Data Preparation

The MOCAP data were cleaned and filled by an experimented operator, which also annotated the repetitions and start/stop, which were defined by the ground force reaction (GFR) stabilization of the subject. For the HD EMG data, the portion of interest of the raw signals was first extracted using the data from the broadcast trigger from the Qualysis Track Manager (Qualisys AB, Göteborg, Sweden, version 2021.2, build 6940). Following this, the HD EMG signals were filtered for baseline wander noise, also called baseline fluctuation. To achieve this, a filter was implemented, based on the work of A. Fasano and V. Valeria [49,50], to which an automatized function of our own design was added to find the optimal lambda for that filter. After this process, the HD EMG signals were filtered for electrocardiogram (ECG) contamination. Indeed, as the EMG was acquired on the trunk region, the electrodes picked up some of the ECG signals. To take care of that issue, a filter based on the work of J. Mak, Y. Hu and K. Luk [50,51] was implemented. After the ECG filtering step, the HD EMG signal was filtered for remaining outliers using the function filloutliers() from MATLAB (MathWorks, Natick, MA, USA). The HD EMG data were then segmented by repetitions. For the data used in the convolutional neural network (CNN), each repetition was resampled 6000 times, to form a 26×10×6000 matrix, when the signals of the 4 patches were concatenated together.

#### 2.4.2. Variables Used

The following variables were chosen to represent the features extracted from the HD EMG and MOCAP data for exploratory analysis, as NSLBP presents attested differences in those variables when compared to the healthy population.

##### Neuromuscular Control Variables

- *Centroid of the HD EMG activity*
  This variable indicates where the EMG activity is concentrated, marking either a specific patch or a group of patches. This variable was chosen as it has already been proven to discriminate between LBP and healthy populations [42], as it yields information about the neuromuscular strategies of the subject.
  The centroid variables used were as follows:
  − Centroid of the right lower back electrode grid, X and Y position;
  − Centroid of the right upper back electrode grid, X and Y position;
  − Centroid of the left lower back electrode grid, X and Y position;
  − Centroid of the left upper back electrode grid, X and Y position;
  − General centroid, X and Y position.
  X and Y are the axes of focus of the location of the centroid: X is the sagittal axis and Y is the transverse axis (corresponding, respectively, to the axes X and Z displayed in Figure 2). The centroids were computed for each repetition and then averaged to determine the centroid value for the movement. This was performed for each subject and for each movement.
  To compute the centroid, the first step was to compute the root mean square (RMS) value of each electrode signal; then, each electrode was mapped as a matrix in which its position corresponded to its actual physical position on the electrode grid. The weighted barycenter of the matrix was then computed for each electrode grid, using the RMS values previously calculated as weight, using the following formula [52,53]:
  (1) $$barycentre=\sum_{i=1}^{N}\left(\frac{\vec{y}}{\sum_{i=1}^{N}y_{i}}\right)\times C_{i}$$
  where, *N* is the number of electrodes, $\vec{y}$ is the mean vector of the RMS values on the transverse axis, and $\vec{C_{i}}$ is the coordinate value of the electrode for the transverse axis $\vec{C}=\left[1,2,\cdots ,8\right]$. The computation for the sagittal axis is the same, but with the mean vector $\vec{x}$ of the RMS value on the sagittal axis and $\vec{C}=\left[1,2,\cdots ,5\right]$. To determine the general centroid, the centroid of each electrode grid was offset by the position of the grid on the back of the person. The [0,0] position corresponds to the position where every grid connects. The general centroid was weighted by the sum of the RMS values of each electrode grid. The processing was inspired by the work of Falla et al. [40].
  The centroid of the EMG activity indicates where the centroid of the contraction is positioned, where most of muscle activity seems to be distributed. This is valuable information, as Sanderson and collaborator showed in their work [42], as LBP subjects tend to have more cranial activation of their lower back muscles, meaning that the muscle activity is distributed more toward the upper regions in comparison to the healthy population.  
- *HD EMG entropy*
  The acquired information can be interpreted, in this case, as a representation of the quality of the command sent by the brain, the command efficiency. This means that the muscle activity is much more localized and less distributed. On the other hand, a high entropy value means that the activity is much more distributed across the recording zone. This variable summarizes information about the neuromuscular strategies of the participants by providing information about the zone of muscular activity. Contrary to the centroid of the activity, which provides information about the general distribution of the muscle activity from a global standpoint, the entropy provides information on the relative distribution of the muscle activity from a structural standpoint.
  The entropy of a signal indicates the amount of information in the signal. The higher the entropy value, the less information the signal contains [54]. Therefore, a low entropy value means that a signal contains a lot of information. EMG information can be interpreted as a representation of the quality—the efficiency—of the command sent by the brain to the muscles. The lower the entropy value, the more the muscle activity is constrained to a specific region and the less it is distributed across the recording zone.
  The entropy variables used were as follows:
  − Entropy of the right lower back electrode grid;
  − Entropy of the right upper back electrode grid;
  − Entropy of the left lower back electrode grid;
  − Entropy of the left upper back electrode grid.
  To compute the entropy of each HD EMG grid, the RMS value of each HD EMG signal of the grid was computed. Then, the entropy of the grid was computed using the Shannon entropy equation [55]:
  (2) $$entropy=\sum_{i=1}^{N}n_{i}^{2}\times \log\left(n_{i}^{2}\right)$$
  where *N* is the number of electrodes and $n_{i}$ is the RMS value of the *i*th electrode. The entropy values were computed for each repetition and then averaged to obtain the entropy value for the movement. This was performed for each subject and for each movement.
  Entropy summarizes information about the neuromuscular strategies employed by the participants by providing information about the distribution of muscular activity across the recording area. On the other hand, the centroid of muscle activity provides information about the general position of the activity. To summarize the difference, the entropy indicates the amount of concentration of the muscle activity, and the centroid of activity indicates the localization of this activity.

##### Variability and Adaptability in the Movement Variables

NSLBP populations have significant differences when it comes to inter- and intra-subject variation [56,57,58]. Therefore, those aspects were integrated in the present work. Inter- and intra-subject variation was assessed as the error against the mean trajectory from the healthy population. Another measure, the entropy of the movement, was also added. This measure indicates the smoothness of the trajectory: the higher the entropy value, the more jittery the movement. This could be interpreted, for a high entropy value, as a movement that requires a lot of readjustment, and for a low entropy value, as a smoother movement. Additionally, for extremely low values of entropy, the movement could even be interpreted as rigid, and unable to adapt to the inevitable small perturbations that a subject faces.

- *Inter-subject Variability*
  − Left shoulder trajectory X-, Y-, and Z-axes inter-variation;
  − Right shoulder trajectory X-, Y-, and Z-axes inter-variation.
  The X, Y, and Z axes correspond to the axes shown in Figure 2. The inter-subject variation, called inter-variation, is the variation in a subject compared to other subjects. First, the average trajectory of the healthy subjects was computed in each axis: X, Y, and Z. All trajectories were re-sampled to be represented by vectors of 100 samples, each before comparison. Following this, for each repetition, the RMS error (RMSE) of the trajectory of the subject against the average healthy trajectory was computed. It was then averaged to obtain one error value, the inter-variation. This was performed for each subject and each movement. The inter-subject variation was normalized by the height of the subject.  
- *Intra-subject Variability*
  − Left shoulder trajectory X-, Y-, and Z-axes intra-variation;
  − Right shoulder trajectory X-, Y-, and Z-axes intra-variation.
  The X, Y, and Z axes correspond to the axes shown in Figure 2. The intra-subject variation, called intra-variation, is the variation in a subject against themselves. First, the average trajectory of the subject was computed in each axis, X, Y, and Z. All trajectories were re-sampled to be represented by vectors of 100 samples each before comparison. Following this, for each repetition, the RMS error (RMSE) of the trajectory of the repetition compared with average trajectory of the subject’s other repetitions was computed. The errors values for each repetition were then averaged to obtain one error value, the intra-variation, for that movement. This was performed for each subject and each movement. The intra-subject variation was normalized by the height of the subject.  
- *Entropy of the movement*
  − Entropy of the left shoulder trajectory, X-, Y-, and Z-axes;
  − Entropy of the right shoulder trajectory, X-, Y-, and Z-axes;
  − Entropy of the hips cluster of markers trajectory, X-, Y-, and Z-axes;
  − Entropy of the T6 vertebrae cluster of markers trajectory, X-, Y-, and Z-axes;
  − Entropy of the C7 vertebrae cluster of markers trajectory, X-, Y-, and Z-axes.
  The X-, Y-, and Z-axes correspond to the axes shown in Figure 2. The entropy for each axis of each shoulder marker was computed following the same logic as for the calculation of the HD EMG entropy, with the difference that the sample entropy [59] was used, as we studied physiological time series signals. The sample entropy was computed using the implementation developed by K. Lee [60].

##### Movement Strategies Variables

The NSLBP population seems to present different movement strategies [21,42,43,61,62,63,64] and a diminished range of movement [62,65], even if those changes are inconsistent across the LBP population.

According to the previous work of Laird et al., 2014 [21], in order to investigate the different movement strategies displayed by the participants, the maximum amplitude and time to maximum amplitude of the participants were used, as well as the maximum angle and time to maximum angle, the latter being used when analyzing the rotation movements.

- *Maximum amplitude of the movement*
  − Maximum amplitude of the left shoulder trajectory, X-, Y-, and Z-axes;
  − Maximum amplitude of the right shoulder trajectory, X-, Y-, and Z-axes;
  − Maximum amplitude of the hips cluster of markers trajectory, X-, Y-, and Z-axes;
  − Maximum amplitude of the T6 vertebrae cluster of markers trajectory, X-, Y-, and Z-axes;
  − Maximum amplitude of the C7 vertebrae cluster of markers trajectory, X-, Y-, and Z-axes.
  The X, Y, and Z axes correspond to the axes shown in Figure 2. The maximum amplitude of each marker and each axis was computed as the maximum absolute difference reached from the position in the first frame. The calculation was performed for each repetition, and then averaged to obtain a value for each subject and each movement. The values were normalized by height.  
- *Time to maximum amplitude*
  − Time to maximum amplitude of the left shoulder trajectory, X-, Y-, and Z-axes;
  − Time to maximum amplitude of the right shoulder trajectory, X-, Y-, and Z-axes;
  − Time to maximum amplitude of the hips cluster of markers trajectory, X-, Y-, and Z-axes;
  − Time to maximum amplitude of the T6 vertebrae cluster of markers trajectory, X-, Y-, and Z-axes;
  − Time to maximum amplitude of the C7 vertebrae cluster of markers trajectory, X-, Y-, and Z-axes.
  The X, Y, and Z axes correspond to the axes shown in Figure 2. The time to maximum amplitude of each marker and each axis was computed as the time it took for the subject to reach the maximum amplitude. The calculation was performed for each repetition, and then averaged to obtain a value for each subject and each movement.
- *Maximum angle and time to maximum angle*
  − Maximum value and time to maximum value of the shoulder angle on the Z-rotation axis;
  − Maximum value and time to maximum value of the angle between the hips and C7 clusters of markers on the X-, Y-, and Z-rotation axes;
  − Maximum value and time to maximum value of the angle between the T6 and C7 clusters of markers on the X-, Y-, and Z-rotation axes.
  The Z-axis corresponds to the axis shown in Figure 2. The maximum angle was computed as the maximum absolute angle reached during movement by the subject. It was only computed for the rotation around the Z-axis. The time to maximum value is the time it took the subject to reach this maximum angle. The values were computed for each repetition, and then averaged to obtain a value for each subject and each movement.

##### Balance and Proprioception Variables

The NSLBP population showcases balance [66,67] and proprioception alterations [68,69,70]. To assess this aspect, we summarized the force plate data in two variables that yield information on the proprioception and balance of the participants.

The proprioception of the participants was assessed via the use of the normalized statokinesigram area [71], and the COP projection of the X- and Y-axes [72]. The balance was assessed by the ground force reaction ratio. This ratio indicates the balance strategy of the subjects and how they distribute their weight in order to perform the movements.

- *Area of the normalized statokinesigram*
  The statokinesigram is the projection on the horizontal plane of the center of pressure (COP) of the subject on the force plates [72]. The COP was measured via the force plate, which recorded the ground force reaction of the subject, and therefore their body sway [73]. Different variables can be extracted from it, which have the potential to yield useful and interesting information, such as the area of the statokinesigram: the ellipse that contains 95% of its values. We did not use 100% of the values in order to increase the robustness of the measure against outliers [74]. One of the drawbacks of the statokinesigram is its high inter- and intra-variation; indeed, repeated measurements showed high intra-day and intra-subject variation [75,76]. To circumvent this issue, normalization of the statokinesigram is recommended in order to improve inter- and intra-reliability in the associated variables [75]. We therefore implemented the self-normalization technique of J. M. de Oliveira [74], using the function that he designed [71]. Once the statokinesigram was normalized, the area of the ellipse encompassing 95% of the statokinesigram value was computed. This was performed for each repetition, those values being averaged to obtain a value for each subject and each movement.
- *Ground force reaction ratio*
  The ground force reaction (GFR) ratio was computed using the following equation:
  (3) $$GFR_{ratio}=\frac{\sum_{n=1}^{N}\frac{leftGFR_{n}}{rightGFR_{n}}}{N}$$
  where *N* is the number of samples and $leftGFR_{n}$ and $rightGFR_{n}$ are the values for, respectively, the left and right GFR of the *n*th sample.
  The GFR ratio was added due to the fact that during data acquisition, two distinct trends were noted: subjects with a relatively equal GFR ratio and subjects with an unequal GFR ratio. We therefore put this variable to the test to see if it has any relevance.

##### Metadata Variables

As we discussed earlier, LBP is a multifactor symptom [3]. In order to reflect this aspect, we used metadata. Age, body mass index (BMI), weight, and height were used as metadata in the clustering analysis. As they are only anthropometric data, they only yielded direct or indirect information about the biophysical and genetic factors.

#### 2.4.3. Deep Neural Network

To categorize people into the Healthy or NSLBP group, we used a linear supervised DNN, using a sigmoid activation function for the concerned layers. The model architecture is detailed in Table 2.

We used the python package keras [77] to build the Sequential() DNN to then train it on the chosen data sets. The optimizer used here is the adam optimizer [78], and the loss function is the categorical_crossentropy [79].

The validation data set was set to be 30% of the total data set. The training set was split using the default value of 75% training data and 25% test data. The batch size was set to 10. We also made sure that the training, testing, and validation sets did not have any common subject in order to avoid information leakage into the model. We started with a model trained on the full data set, without the spine data, to maximize the amount of data points at our disposal. This model was created as a null model to assess our supposed maximum classification accuracy. After that, the model was trimmed by domain:

- Anthropometric;
- Biomechanics;
- Neuromuscular control;
- Balance.

Table 3 details the variables used in the different models.

This was carried out to assess the potential of the information in each domain. Some domains were further divided in order to gain a more detailed look into them.

In addition to the deep neural network, a convolutional neural network was used to classify the subjects using the full HD EMG data. The CNN architecture is displayed in Table 4.

The convolutional neural network was fed HD EMG data previously cleaned and divided by repetitions. To keep the complexity of the CNN model to a minimum, we only focused on the data from the **Back Flexion Preferred Speed** movement.

#### 2.4.4. Factor Analysis

Factor analysis (FA) is a variant of the principal component analysis. It differs from the latter in the construction of the correlation matrix used to compute the components and find the factors of those components. An R-type exploratory analysis was run for each movement in the protocol to look for an “NSLBP” component and study its factor loadings [80]. The technique used here for the R-type analysis was the maximum likelihood method developed by K. Joreskog and collaborator [81]. The mathematics of this method are fairly complex, so we will not detail them here, but we recommend the work of S. Mulaik [82] for the curious reader. This technique was developed by A. Comrey [83] and H. Harman [84]. The component rotation algorithm used is the varimax one in order to simplify the structure found by the FA, as the simpler structure is thought to be the optimal result [85,86]. We used the R function fa() from the R package psych to run the FA analysis. We recommend that the reader looking for more details on FA read the book “Easy Guide to Factor Analysis” by P. Kline [80].

We used as few variables as possible, while still trying to tackle most of the aspects of NSLBP to match the factor analysis (FA) requirements [80]. Optimally, FA is performed on samples with a size of at least 100 occurrences, with a ratio of 20:1 subject to variables, but we were not able to meet these requirements, and therefore had to compromise. Only one axis was selected for every FA performed to limit the number of dimensions fed to the FA. The number of dimensions was limited, and only a couple of variables for each of the domains were kept. The number of components was selected via the function nScree() [87] from the R package nFactors [88]. The number of components was the rounded mean result of the different tests run using the nScree() function: Eigenvalues, Parallel Analysis, Optimal Coordinates, and Acceleration Factor.

The variables used for the FA were the following:

- Age;
- Group: Healthy/NSLBP;
- Sex: Male/Female;
- Body mass index (BMI);
- Maximum amplitude of the left shoulder trajectory (max. ROM);
- Time to maximum amplitude of the left shoulder trajectory (time to max. ROM);
- Entropy of the movement of the left shoulder trajectory (traj. entropy);
- Inter-subject variation in the left shoulder trajectory (traj. inter-var.);
- Intra-subject variation in the left shoulder trajectory (traj. intra-var.);
- Entropy of the EMG of the right lower back (EMG entropy l. low back);
- Entropy of the EMG of the left lower back (EMG entropy r. low back);
- Y position of the total back EMG centroid (EMG centroid Y pos.);
- GFR ratio.

For readability of the result, the GFR ratio was sightly changed so that: $GFR_{FA}=\left|GFR\right|-1$. This was done in order to have a balanced ratio on zero and so that changes were not side oriented, making results easier to read, compare, and interpret.

For the variables related to shoulder movement, only one axis and one side were chosen in order to limit the number of variables used to meet the factor analysis requirements for optimal behavior. We chose the axis where the most movement was detected with regard to the movement performed so as to stay as relevant as possible:

- Y-axis for:
  − Back extension, maximum and preferred speed;
  − Trunk rotation, left and right, maximum and preferred speed.
- Z-axis for:
  − Back flexion, maximum and preferred speed;
  − Lateral flexion, left and right, maximum and preferred speed.

For the rotation movements, the maximum angle on the Z-axis and the time to maximum angle on the Z-axis were used instead of the maximum amplitude and time to maximum amplitude of the trajectory.

In our analysis, the following categorical values were simplified in the following way:

- Group:
  − Healthy: 0;
  − NSLBP: 1.
- Sex:
  − Male: 0;
  − Female: 1.

The critical correlation values were assessed using Student’s t-test [89,90]. With our sample size of 46, the critical correlation values are as follows:

- p=0.05 : c=0.29;
- p=0.01 : c=0.38;
- p=0.005 : c=0.41;
- p=0.001 : c=0.47.

## 3. Results

### 3.1. Deep Neural Network

#### 3.1.1. Full Model

This model was trained on the whole set of variables, except for the spine data, so as to maximize the number of data points available. This full model was made to have some sort of a null model, in order to see if it was already possible to categorize healthy and NSLBP subjects from our data set.

After 100 epochs, the model reached an accuracy of 99.88% on the test set and 93.30% on the validation set, showing signs of significant, but not dramatic, overfitting. This model attests that the data acquired contain valuable information about NSLBP.

#### 3.1.2. Anthropometric Model

This model focused on the anthropometric data of the subjects and was trained for 300 epochs with the following variables:

- Age;
- BMI;
- Height;
- Weight;
- Sex.

This model was trained to show how important the impact of only the anthropometric variables was on the capacity to classify the subjects, and therefore, the strength of their link to NSLBP. Indeed, the model, after training, reached a precision score of 92.84% on the test set and 94.40% on the validation set.

A trimmed version of this model, without the weight and the height variables, was also trained and reached a precision score of 94.40% on the test set and 98.19% on the validation set.

While training these models, we achieved a very high accuracy result despite the fact that we solely fed them basic anthropometric data. These results were obtained whilst not displaying any obvious sign of overfitting.

#### 3.1.3. Biomechanical Model

This model was built with biomechanics data and trained for 400 epochs, using the following variables:

- Maximum amplitude of the left shoulder trajectory in the X-, Y-, and Z-axes;
- Time to maximum amplitude of the left shoulder trajectory in the X-, Y-, and Z-axes;
- Entropy of the movement of the left shoulder in the X-, Y-, and Z-axes;
- Intra-subject variation in the movement of the left shoulder in the X-, Y-, and Z-axes;
- Inter-subject variation in the movement of the left shoulder in the X-, Y-, and Z-axes;
- Position of the barycenter of the feet in the X-, Y-, and Z-axes;
- Movement performed;
- Speed of the movement.

After training, we achieved a precision of 83.05% for the test set and 84.47% for the validation set after 90 epochs, but it started overfitting after that.

The same model was trimmed down and trained with only very basic information:

- Maximum amplitude of the left shoulder trajectory in the X-, Y-, and Z-axes;
- Time to maximum amplitude of the left shoulder trajectory in the X-, Y-, and Z-axes;
- Position of the barycenter of the feet in the X-, Y-, and Z-axes;
- Movement performed;
- Speed of the movement.

After being trained for 398 epochs, the model reached an accuracy of 88.29% on the test set and 88.45% on the validation set before overfitting.

That same model was trained anew, this time with the variable normalized by subject height. Indeed, as shoulder displacement in space is correlated with subject height, chances are that the subject interferes with the relative displacement, tempering our results in a negative way. Normalizing by subject height would allow us to focus on the actual relative movement of the subject in space, which should yield more accurate information and therefore better results. This time, after 194 epochs, we reached a maximum accuracy of 87.40% on the test set and 92.96% on the validation set, before the performance deteriorated and began overfitting.

These results show us that we can gain a lot of information from just subjects’ motion information. Indeed, while feeding the model with only limited and basic information about the movement of the subjects, we still achieved categorization with a very high accuracy score.

#### 3.1.4. Neuromuscular Model

This model was trained using all the variables related to the neuromuscular aspect of the subject:

- Centroid of the EMG activity of each of the four patches of electrodes;
- Global centroid of the EMG activity of the four patches of electrodes;
- EMG entropy of each of the four patches of electrodes;
- Movement performed;
- Speed of the movement.

After being trained for 15 epochs, the model reached a maximum accuracy of 83.27% on the test set and 88.07% on the validation set before performance deteriorated and began overfitting. These results are relatively high, so we attempted to improve them by trimming down the model.

A model accounting only for the entropy of the four patches was trained. This model reached a maximum accuracy of 76.21% on the test set and 87.30% on the validation set after 104 epochs, after which the performance stayed stable for around 80 epochs before it degraded and overfit; indeed, clear signs of overfitting started to appear around 375 epochs.

Another model accounting for the centroid of the four patches was trained. This model reached a maximum accuracy of 71.13% on the test set and 82.69% on the validation set in 285 epochs. Here, we present details about the model accuracy and loss. The valuable data seem to be the centroid of the EMG, or at least, it seems to be less susceptible to the noise that could not be filtered out of the EMG. The behavior of the loss seemed relatively unexceptional. The behavior of the accuracy lets us consider the potential local minima trap, which changes our expectation concerning the extrapolation capacity of the model on other data sets. Indeed, the accuracy starts off with a very high value and stays extremely stable, exhibiting a non-typical “stuck”-like behavior, but it tends to decrease quickly after more epochs.

These results show us that we can gain a lot of information just from subjects’ basic neuromuscular information. Indeed, while feeding the model solely with limited and basic information about the neuromuscular control of the subjects, we still achieved categorization with a decent accuracy score, even if some overfitting issues appeared. As this does not happen on the full neuromuscular data set, this might indicate that the variables from the neuromuscular domain which we used could be complementary, and the variables are affected in synergy with each other by NSLBP.

Following this, we trained the CNN model using the preprocessed HD EMG data. The accuracy and loss of the model were found, respectively. The model reached a maximum accuracy of 100% on the test set in only a couple of epochs but only reached a low and unstable accuracy score. This result puts in perspective the lack of a training sample. The fact that the model could learn such a high accuracy on the test set is encouraging, as it means that there is something to learn from the data, but the low validation accuracy results remind us that we cannot infer whether the pattern learned by the model is correlated to NSLBP or not.

#### 3.1.5. Balance Model

This model used variables related to the balance and proprioception of the subjects:

- Area of the normalized statokinesigram;
- GFR ratio;
- Movement performed;
- Speed of the movement.

After being trained for 68 epochs, the model reached a maximum accuracy of 68.90% on the test set and 74.76% on the validation set, after which the model overfitted and performance deteriorated significantly. Here, we present details about the model accuracy and loss. The model shows the same “stuck”-like behavior on its accuracy metrics as the centroid model. This added to the overfitting behavior acknowledged by the loss value, warning us not to draw strong conclusions from that model’s results, which might be due to the lack of sample and lucky local minima.

#### 3.1.6. Summary

Table 5 below summarizes the results from the DNN training.

### 3.2. Factor Analysis

#### 3.2.1. Back Extension, Maximum

Using the Z-axis as the axis of interest, the fourth component loaded the group factor at a significance level of 0.370 (*p* < 0.05). The loadings associated with it are:

- Time to maximum amplitude of the left shoulder trajectory: 0.786 (*p* < 0.001);
- Entropy of the movement of the left shoulder trajectory: −0.645 (*p* < 0.001);
- Y position of the total back EMG centroid: 0.338 (*p* < 0.05).

Age is also close to being significant, with a loading of 0.280 (*p* > 0.05). It seems that people with NSLBP move at a slower rate than their healthy counterparts, and their movement is less “noisy”. The latter could be interpreted either as a more efficient movement, or a more rigid movement. We interpret it as a more rigid movement due to the behavior of the subjects afflicted by the symptom, which was much more cautious [57,91,92], an interpretation corroborated by the longer time it took them to complete the movement. This could also be, in part, due to a lack of adaptability to the movement conditions. We can see that the EMG activity is distributed more cranially among the NSLBP subjects, as seen by Sanderson and collaborator [42] during other tasks.

We can note the large loading of age in the first factor, and the significant loading of age in the other factor, which could indicate that age has a tremendous impact on the movement itself [93,94].

#### 3.2.2. Back Extension, Preferred

Using the Z-axis as the axis of interest, the second component loaded the group factor at a significance level of 0.292 (*p* < 0.05). The loadings associated with it are:

- Age: 0.381 (*p* < 0.01);
- Time to maximum amplitude of the left shoulder trajectory: 0.958 (*p* < 0.001);
- Entropy of the movement of the left shoulder trajectory: −0.736 (*p* < 0.001);
- Y position of the total back EMG centroid: 0.338 (*p* < 0.01).

We can draw the same conclusion than when the movement is performed at maximum speed, the difference caused by age seems to have even more correlation to the NSLBP component.

As the NSLBP factor is not loaded significantly, FA was conducted on the Y- or X-axis, but with no better results. Therefore, we do not dwell upon it in this article.

#### 3.2.3. Back Flexion, Maximum

Using the Z-axis as the axis of interest, the second component loaded the group factor at a non-significant level: 0.237 (*p* > 0.05), with the associated significant loadings:

- Maximum amplitude of the left shoulder trajectory: 0.582 (*p* < 0.001);
- Time to maximum amplitude of the left shoulder trajectory: 0.440 (*p* < 0.005);
- Inter-subject variation in the left shoulder trajectory: −0.324 (*p* < 0.05);
- Intra-subject variation in the left shoulder trajectory: −0.923 (*p* < 0.001).

These loadings seem to attest the different movement strategies between healthy and NSLBP subjects. While they have a greater range of motion, the NSLBP subjects take longer to achieve the movement. Of course, the inter-subject component loading is a direct expression of the different movement strategies between healthy and NSLBP subjects. At the same time, significant loading was seen in the intra-subject variation, meaning that NSLBP subjects do not adapt their movement much with regard to new starting conditions [57]. Nonetheless, as the loading was non-significant for the group component, those results are not to be taken at face value, as examined in the associated discussion.

Factor analysis was conducted on the Y and X axes, but did not yield any significant loading on the group component and was, therefore, not discussed here.

#### 3.2.4. Back Flexion, Preferred

Using the Z-axis as the axis of interest, the second component loaded the group factor at a significant level: 0.986 (*p* < 0.001). However, no other factors significantly loaded this component. We found age (0.148; *p* > 0.05), BMI (0.240; *p* > 0.05), time to maximum amplitude (0.270; *p* > 0.05), the position of the EMG centroid of activity on the Y-axis (0.186; *p* > 0.05), and the GFR ratio (−0.230; *p* > 0.05), all loading at a low and significant level. A new factor analysis was conducted, this time using the Y-axis as the axis of interest.

Using the Y-axis as the axis of interest, the sixth component loaded the group factor at a significant level: 0.520 (*p* < 0.001), with the associated significant loadings:

- BMI: 0.405 (*p* < 0.01);
- Entropy of the movement of the left shoulder trajectory: 0.553 (*p* < 0.001);
- Entropy of the EMG of the left lower back: -0.420 (*p* < 0.005);
- Y position of the total back EMG centroid: 0.538 (*p* < 0.001).

BMI seems to have a great influence on the component for this movement. This result is not extremely surprising, as the NSLBP population tends to be associated with a higher mean BMI than their healthy counterparts [95,96]. As with the back extension movements, the entropy of the movement of the left shoulder trajectory seems to indicate that the movement produced by the NSLBP subjects is more rigid, with less fine-tuned adaptations. The entropy of the EMG of the left lower back seems to indicate muscle activity that is less localized and noisier. This is associated with the value of the general centroid position in the Y-axis, indicating a higher activation of the upper portion of the lower back, which has been shown in other work [42]. The maximum amplitude variable does not load the group component, a surprising finding regarding the literature.

#### 3.2.5. Lateral Flexion Left, Maximum

Using the Z-axis as the axis of interest, the second component loaded the group factor at a significant level: 0.387 (*p* < 0.01), with the associated significant loadings:

- Time to maximum amplitude of the left shoulder trajectory: 0.504 (*p* < 0.001);
- Inter-subject variation in the left shoulder trajectory: 0.872 (*p* < 0.001);
- Intra-subject variation in the left shoulder trajectory: 0.299 (*p* < 0.05);
- GFR distribution ratio: −0.481 (*p* < 0.001).

These loadings can be interpreted as NSLBP subjects producing movements significantly different to those of their healthy counterparts. In addition, NSLBP subjects seems to distribute their weight more equally between each foot [43]. This could be seen as a lack of adaptation to the movement, limiting their performance, in order to maximize the instantaneous feeling of safety and control, for example, by not working with the momentum of the movement. They also have a higher variation between repetition trajectories, something that seems to go against findings in other movements. Nonetheless, this does not go against the literature, which reports much intra-subject variation in the NSLBP population [56]. It could be hypothesized that the adaptations to NSLBP do not affects movements in the same way, as the different results per movement in our analysis seem to indicate. Again, the time to maximum amplitude is significantly higher in people with NSLBP than their healthy counterparts.

#### 3.2.6. Lateral Flexion Left, Preferred

Using the Z-axis as the axis of interest, the third component loaded the group factor at a non-significant level: 0.273 (*p* > 0.05), with the associated significant loadings:

- Age: 0.856 (*p* < 0.001);
- Sex: −0.332 (*p* < 0.01);
- BMI: 0.447 (*p* < 0.005);
- Time to maximum amplitude of the left shoulder trajectory: 0.465 (*p* < 0.005);
- Entropy of the movement of the left shoulder trajectory: −0.411 (*p* < 0.005);
- Inter-subject variation in the left shoulder trajectory: 0.473 (*p* < 0.001);
- Intra-subject variation in the left shoulder trajectory: 0.525 (*p* < 0.001);
- GFR distribution ratio: −0.558 (*p* < 0.001).

The loading for the variable group was not significant, so no strong conclusions can be drawn from those results. A new factor analysis was conducted, but this time using the Y-axis as the axis of interest.

Using the Y-axis as the axis of interest, the third component loaded the group factor at a significant level: 0.368 (*p* < 0.05), with the associated significant loadings:

- Age: 0.716 (*p* < 0.001);
- Sex: −0.403 (*p* < 0.01);
- BMI: 0.395 (*p* < 0.01);
- Time to maximum amplitude of the left shoulder trajectory: 0.622 (*p* < 0.001);
- Entropy of the movement of the left shoulder trajectory: −0.380 (*p* < 0.01);
- Inter-subject variation in the left shoulder trajectory: 0.618 (*p* < 0.001);
- GFR distribution ratio: −0.622 (*p* < 0.001).

We can see that the NSLBP group tends to perform movements at a slower pace, and more rigidly, than the healthy counterparts. Notably, as with the results for the Lateral Flexion Left, Maximum, a higher variation between repetitions in the NSLBP group can be seen. Again, NSLBP subjects tend to be correlated with a more balanced distribution of the GFR. One interesting thing to note is the massive importance of the anthropometric variables on this movement: age, 0.856 (*p* < 0.001); sex, −0.332 (*p* < 0.05); and BMI, 0.447 (*p* < 0.005). From this, we concluded that anthropometric variables have a great impact on this movement, but also that male subjects, those with a higher BMI, older subjects, or those with a combination of these factors tend to express the adaptations to NSLBP more dramatically than others.

#### 3.2.7. Lateral Flexion Right, Maximum

Using the Z-axis as the axis of interest, the third component loaded the group factor at a non-significant level: 0.221 (*p* > 0.05), with the associated significant loadings:

- Age: 0.326 (*p* < 0.05);
- Time to maximum amplitude of the left shoulder trajectory: 0.614 (*p* < 0.001);
- Entropy of the movement of the left shoulder trajectory: −0.418 (*p* < 0.005);
- Entropy of the EMG of the right lower back: −0.585 (*p* < 0.001);
- Y position of the total back EMG centroid: 0.429 (*p* < 0.005).

The loading for the variable group was not significant, so no strong conclusions can be drawn from those results. A new factor analysis was conducted, this time using the Y- or X-axis as the axis of interest, but no component loaded the group variable. Strangely, this problem did not arise when the same movement was performed at the same speed but on the other side. Nonetheless, we can see that the factors loading the non-significant NSLBP component are still similar to the results from other movements, but as the loading on the group factor was never significant for any of the component, we did not dwell upon those results.

#### 3.2.8. Lateral Flexion Right, Preferred

Using the Z-axis as the axis of interest, no component really loaded the group variable. The second component, 0.111 (*p* > 0.05), and third component, 0.154 (*p* > 0.05), did, but at very low levels. Due to the non-significant value of the correlation with the group variable, no conclusion can be drawn, and therefore, a new factor analysis was conducted, this time using the Y-axis as the axis of interest.

Using the Y-axis as the axis of interest, the fourth component loaded the group factor at a non-significant level: 0.281 (*p* > 0.05), with the associated significant loadings:

- Age: 0.786 (*p* < 0.001);
- Sex: −0.493 (*p* < 0.001);
- BMI: 0.464 (*p* < 0.005);
- Time to maximum amplitude of the left shoulder trajectory: 0.397 (*p* < 0.01);
- Inter-subject variation in the left shoulder trajectory: 0.460 (*p* < 0.005).

As no component loaded the group at a significant level, a new factor analysis was conducted, this time using the X-axis as the axis of interest.

Using the X-axis as the axis of interest, the fourth component loaded the group factor at a significant level: 0.308 (*p* < 0.05), with the associated significant loadings:

- Age: 0.442 (*p* < 0.005);
- BMI: 0.818 (*p* < 0.001);
- Y position of the total back EMG centroid: 0.387 (*p* < 0.01);
- GFR distribution ratio: −0.357 (*p* < 0.05).

We can see that, mainly, it is the anthropometric variables that load the NSLBP component. We again see that NSLBP subjects tend to be correlated with a more balanced distribution of the GFR, and that their muscle activity seems to be distributed more cranially.

#### 3.2.9. Trunk Rotation Left, Maximum

Using the Z-axis as the axis of interest, the fifth component is loaded the group factor at a significant level: 0.531 (*p* < 0.001), with the associated significant loadings:

- Age: 0.319 (*p* < 0.05);
- Maximum angle displacement on the Z-axis for the left shoulder: 0.547 (*p* < 0.001);
- Inter-subject variation in the left shoulder trajectory: 0.356 (*p* < 0.05);
- GFR distribution ratio: −0.300 (*p* < 0.05).

Age seems to be a significant factor, which is not surprising at this point. Here, a counter-intuitive result was found: the NSLBP group seems to be associated with an overall greater maximum angle of rotation than that of their healthy counterparts. As excepted, NSLBP subjects are associated with a different movement trajectory than healthy subjects. We also see that they tend to distribute their weight more evenly between their feet than the healthy population.

#### 3.2.10. Trunk Rotation Left, Preferred

Using the Z-axis as the axis of interest, the third component loaded the group factor at a significant level: −0.378 (*p* < 0.05), with the associated significant loadings:

- GFR distribution ratio: 0.943 (*p* < 0.001).

Here, the only variable that loaded the group was the GFR distribution ratio, showing that the healthy population seems to have an extremely uneven weight distribution between their feet.

The main peculiarity here is that we have a second component loading the group factor, close to a significant level, toward the NSLBP group rather than the Healthy group, as in in the third component. We have, therefore, a “Healthy” component and an “NSLBP” component in the same movement analysis, which is quite interesting. For the curious reader, the NSLBP component loads are Age at 0.543 (*p* < 0.001) and BMI at 0.715 (*p* < 0.001), which again present the strong relation between the anthropometric variables and NSLBP. The trajectory of the movement produced by NSLBP subjects was significantly different than the ones from the Healthy group, with a loading of 0.305 (*p* < 0.05) of the inter-movement variation factor. In addition, the centroid of the EMG activity seems to be more cranial for the NSLBP group, with a loading of 0.421 (*p* < 0.005). Interestingly, the “Healthy” component does not load the anthropometric data significantly, in stark contrast with the NSLBP components found in the other movements. Nonetheless, as the loading on the NSLBP component does not reach a significant level, no hard conclusion can be drawn.

#### 3.2.11. Trunk Rotation Right, Maximum

Using the Z-axis as the axis of interest, the second component loaded the group factor at a non-significant level: −0.217 (*p* > 0.05), with the associated significant loadings:

- Age: −0.390 (*p* < 0.01);
- Maximum angle displacement on the Z-axis for the left shoulder: 0.308 (*p* < 0.05);
- Time to maximum angle displacement on the Z-axis for the left shoulder: −0.950 (*p* < 0.001);
- Entropy of the movement of the left shoulder trajectory: 0.680 (*p* < 0.001);
- Entropy of the EMG of the right lower back: 0.519 (*p* < 0.001).

Unusually, the component loading the group factor does so toward the Healthy group. The healthy population is associated with a bigger amplitude of movement, as well as a much faster movement speed. The higher entropy of the movement trajectory seems to show a movement that presents more micro-adaptations, which could be interpreted as a higher correction of the movement [57]. The higher EMG entropy associated with the component seems to indicate a more diffuse muscular activity of the lower back region [42]. Unlike when performing the movement at maximum speed, the component is associated with a lower average age this time. Interestingly, the first and third components also loaded the group factor toward NSLBP, albeit to a non-significant level. Nonetheless, as the loading of the group variable on the components was not significant, no conclusions can be drawn from those results. A new factor analysis was conducted, this time using the Y-axis as the axis of interest.

Using the Y-axis as the axis of interest, the first component loaded the group factor at a significant level: 0.321 (*p* < 0.05), with the associated significant loadings:

- Age: 0.532 (*p* < 0.001);
- BMI: 0.349 (*p* < 0.05);
- Time to maximum amplitude of the left shoulder trajectory: 0.746 (*p* < 0.001);
- Entropy of the movement of the left shoulder trajectory: −0.594 (*p* < 0.001);
- Intra-subject variation in the left shoulder trajectory: −0.424 (*p* < 0.005);
- Y position of the total back EMG centroid: 0.405 (*p* < 0.01);
- Entropy of the EMG of the right lower back: −0.597 (*p* < 0.001).

From the factor’s loadings, NSLBP subjects seem to be associated with slower movement, as the very high loading of the time to maximum amplitude of the left shoulder trajectory indicates, as well as the lower entropy of the left shoulder trajectory, which could be due to a more rigid movement with less micro-adjustments by the NSLBP subjects. Interestingly, NSLBP seems to be associated with a lower entropy of the EMG signal in this movement, which could indicate a muscular activation that is more localized than in healthy participants. At the same time, the intra-subject variation seems to be lower in the NSLBP group, possibly indicating a lack of adaptation capability between repetitions. The NSLBP component is associated with a higher BMI and older age.

#### 3.2.12. Trunk Rotation Right, Preferred

Using the Y-axis as the axis of interest, the third component loaded the group factor at a significant level: 0.387 (*p* < 0.01), with the associated significant loadings:

- Age: 0.494 (*p* < 0.001);
- Maximum angle displacement on the Z-axis for the left shoulder: 0.587 (*p* < 0.001);
- Time to maximum angle displacement on the Z-axis for the left shoulder: −0.345 (*p* < 0.05);
- Inter-subject variation in the left shoulder trajectory: 0.373 (*p* < 0.05);
- Y position of the total back EMG centroid: 0.412 (*p* < 0.005).

Aside from the age variable loading, we see that NSLBP subjects tend to showcase a larger amplitude of movement and faster speed of execution. The higher speed of execution and larger amplitude are a bit counter-intuitive. This could be a way to alleviate discomfort by performing the movement faster in order to be done with it. The larger amplitude may be a side effect of the momentum created by the increased speed, which would be more difficult for NSLBP subjects to control without compromising their spine integrity or pain level. Nonetheless, this is still counter-intuitive looking back on the results from the same movement performed at maximum speed. In addition, they displayed a higher difference in movement trajectory compared to their healthy counterparts. Additionally, their muscle activity is more cranially distributed.

#### 3.2.13. Summary

Table 6 and Table 7 summarize the results from the factor analysis for the movements at preferred and maximum speed.

## 4. Discussion

Using the DNN, it was shown that the variables chosen yielded a substantial amount of information about the status of the subject, healthy or NSLBP. The subdivision into domains showed that information was not constrained to some domains only, but was distributed across them. It should be noted that the information power was equally distributed, aside from the force plate data. In the context of clustering the NSLBP population, those results align with the consensus of considering the NSLBP symptom as a multi-factorial problem; therefore, the clustering solution should itself rely on data that reliably represent the five main domains driving the NSLBP prognosis [1,3].

It should be mentioned that, whilst most of the variables yielded relatively high accuracy results, this was not the case for the balance model or the CNN model. Stabilometry data are known to present significant differences between NSLBP and healthy subjects [69], but to present accurate and reliable results, stabilometry data should abide to certain standards, one such standard being that data should be acquired from a sample of at least 90 s [97]. This criterion is rarely met when a subject is performing a dynamic movement, which lasts only a few seconds. On the other hand, using a static standing recording to cluster the NSLBP population would overlook some of the neuromuscular and biomechanical differences that arise during dynamic tasks [21,42,57,63,91]. Looking at the counterperformance of the balance model, especially in light of the relatively high accuracy of the biomechanical and neuromuscular models, it seems that, when trying to cluster the NSLBP population, it is better to focus on dynamic movements.

Concerning the subpart performance of the CNN, we suspect that the issue is a methodological one and not a data-driven one. Beyond the problem of limited data points, studying the HD EMG signals from the subject performance as a “simple” image using CNN might overlook some of the temporal relations present in those signals that cannot be grasped by a pure CNN. That temporal aspect might be of great importance for classification. Therefore, in order to use HD EMG signals without any feature extraction, we recommend turning to solutions that also take into account the temporal resolution of those signals via the use of more complex models, such as support vector machine (SVM), for example, ref. [98], or a combination of CNN and SVM, which could be more adapted for such complex data [99].

In addition, finding deep learning techniques yielding a good performance, even with limited data, while clustering the population is still a hard and complex task, which is a testament to the complexity or subtlety in the relation between the variables and NSLBP. As with the problem we encountered with the CNN, those results might be a hint to push toward tools that can detect such complex relationships, or to find higher-level variables that would encompass the NSLBP domains of interest while reducing the degree of freedom of the clustering problem in order to make it simpler for classic techniques.

The results from the FA show us that not all variables and domains of variables are equal in regard to different movements. Depending on the movement performed, the amount of information related to NSLBP varies across variables, and probably across domains of variables, too. In light of those results, we recommend focusing on single-movement-based classification, and keeping the task/movement simple, so as to prevent the complexification of the clustering task. Indeed, if different simple movements are strongly linked to different variables, it could be hypothesized that a complex movement, being a mix of simple movements, would see the number of strongly related variables add up, distributing the significant information across those variables, thus creating a more complex clustering problem. In addition to focusing on a single simple movement for acquiring data, the focus should be placed on relevant associated variables. The benefit would be twofold: simpler study protocols, and if working solutions are found from them, easier clinical application.

From our point of view, trunk flexion could be a strong candidate for the movement of choice, as it is a well-studied movement [21]. However, this movement also has the particularity of displaying the flexion relaxation phenomenon: the reduction in paraspinal muscle activity at the maximum trunk flexion. This phenomenon is known to present differences between the NSLBP and healthy populations [100]. Another candidate is the trunk rotation. Indeed, this movement had different components loading in both the healthy and NSLBP directions, which could mean that this movement has an increased discriminatory capacity compared to the others.

Even using restricted data sets, the DNN models showed, most of the time, high accuracy results despite the complexity of the NSLBP symptom and the variation in its expression among afflicted subjects [1,3,21]. In light of this, the case could be made that the tools used to study NSLBP and its clustering need to be rethought, in order to fit the need to find complex and subtle relationships among the data, something that deep learning tools excel at [101,102], where classic clustering techniques face difficulties, which can be harder to address [103].

To elaborate on the previous statements, a case could be made for the possibility of the reliable “meta data clustering” for NSLBP. By meta data, we refer to high-level variables related to the five main domains driving NSLBP prognosis: biophysical, comorbidities, social, psychological, and genetic. Indeed, regarding meta data, in the present exploratory work constrained to the biophysical domain via the anthropometric data, two points can be made: concerning their use in DNN, the models were able to classify, with extreme accuracy, NSLBP and healthy subjects, and concerning their use in FA, they consistently loaded each movement, even though they did so via different variables, on the group factor. Considering such results, a case could be made for the search of a “meta clustering” method, relying on high-level data from the main domains of interest in order to subgroup the NSLBP population. This could provide, for little cost and complexity, a framework that is easy to use clinically. This has already attempted, for example, by the Quebec task force [30], but it failed, maybe because some domains were overlooked. Therefore, the case for meta data or higher-level variables should not be discarded, as they might provide us with a framework allowing us to “summarize” the complex relationships between domains linked to NSLBP and ease the complexity of the task of clustering NSLBP. This could prevent the need for more complex modeling tools or solutions in general, as long as all the main domains influencing the NSLBP prognosis are represented [96,104,105], and the probable effect that the dose/response of the time experiencing NSLBP has on the related adaptations is taken into account [106].

We end this discussion by sharing with the reader a daring thought. In addition to being relatively low cost and easy to test, this last hypothesis yields one more reason to focus on this topic. If actually proven valid, this could push us to ask ourselves: Could NSLBP be a generic symptom that is expressed differently in every individual due to each unique profile in the main domains? While still being in accordance with the consensus about what influences the prognosis of NSLBP, this is a daring question, but one which could lead to extremely interesting perspectives in research and directly in clinical applications [1,3]. Even if daring, this hypothesis is not ungrounded; biological factors are known to be directly linked to NSLBP [95], as are genetic factors [107,108,109]. In addition, it has been shown that health is significantly impacted by the socioeconomic status of an individual [110,111,112,113], and psychological factors have been shown to hinder recovery [114]. These are all parts of the main domains known to impact NSLBP prognosis. If factually proven, this hypothesis would explain the extreme diversity in the NSLBP population: indeed, it is extremely difficult to recruit population samples that all showcase the same profile on the five main domains. Therefore, we can easily suppose that all NSLBP samples studied so far differ in some domain, even if the researchers have made sure that they present homogeneous profiles regarding one of the domains, usually the biophysical one. The rest are usually not controlled, such as the psychological or socioeconomic domains. The heterogeneity of the main factors in the population sample drives the expression of NSLBP in different directions, accordingly to the different subjects’ profiles, which therefore explains the extreme variation in this population and the studied samples and the vastly different reactions to rehabilitation protocols.

### Limitations

One of the two major limitations of this work to be acknowledged is the lack of data points. Our intention was to acquire data on 30 participants for each group, but the COVID-19 pandemic created unforeseen issues and constraints, both logistical and operational. This resulted in an unbalanced subject distribution between groups, which potentially diminished the quality of the results and limited their extrapolation. In addition, the complexity of the MOCAP model used also prevented us taking full advantage of the data acquired, due to the lengthy process of MOCAP data cleaning, which could not fit in our time constraints due to the high number of different movement tasks performed.

Our project focused on continuous variables as we think that the models using them will yield results closer to the clinical reality. Learning more about them and their relationships with NSLBP is of great value, but is also more complex, and more data points and a greater population diversity are required. Care should be taken concerning the extrapolation of those results to the actual population, and they should be used only as guidance, and not hard-set rules to be followed by the book. Especially for FA, the number of variables that could be investigated was limited by our sample size [80].

Nonetheless, the FA and DNN results present, respectively, strong significance and strong accuracy. Therefore, the use of continuous variables should not be discarded in preference of categorical ones when investigating NSLBP, especially when it comes to clustering the NSLBP population for clinical purposes. As categorical clustering models have not yet proven effective in the field [29,115,116] there is a need to develop new solutions.

## 5. Conclusions

This study findings can be summarized in the following points:

- Higher-level data, or “meta data”, linked to the main domains influencing NSLBP prognosis should receive more attention when attempting to cluster the NSLBP population, and no domains should be discarded (a case could be made for the genetic domain due to the complexity of studying it);
- Dynamic conditions used to acquire data in order to study the clustering of the NSLBP population should be kept as simple as possible in order to prevent the complexification of the clustering task;
- As the importance of relationships with NSLBP for each variable is dependent on the condition performed, care should be taken when choosing which variables to examine for each of the conditions studied. Exploratory work should be considered before attempting to cluster a population, and the associated results should be shared along with the clustering results;
- Back flexion and trunk rotation seem to be the ideal movements to be used as the dynamic conditions of choice for data acquisition;
- Tools that have the capacity to detect and model complex and subtle relationships should be prioritized. Great importance should be placed on the choice of data analysis and tool frameworks used to process data from NSLBP when attempting to cluster populations.

Concerning this last point, the focus on higher-order variables linked to the domains influencing NSLBP prognosis should not be disregarded, as they could allow researchers to study more fundamental and common adaptations of NSLBP and its expression, and for the clinicians to apply this knowledge straight away into their practice to help develop personalized and more effective care protocols in accordance with the profile of their NSLBP patients, without the need for new or expensive equipment.

## Figures and Tables

**Table 1:** Population characteristics.

Population	Age (Year)	Weight (kg)	Height (cm)	BMI
General	31.2 ± 9.2	74.4 ± 13.8	172.9 ± 8.5	24.9 ± 4.3
Healthy	29.6 ± 9.1	69 ± 12.7	172.5 ± 8	23.1 ± 3
NSLBP	31.8 ± 9.3	76.7 ± 13.8	173 ± 8.9	25.6 ± 4.6

**Table 2:** General deep neural network architecture. The input layer had an architecture dependent on the input data.

Layer (Type)	Output Shape	Nb of Parameters
input_1 (Dense)	(None, X)	759
dense_1 (Dense)	(None, 128)	1536
dropout (Dropout 0.5)	(None, 128)	0
dense_2 (Dense)	(None, 36)	4644
dense_3 (Dense)	(None, 1)	37

**Table 3:** Variables used in each model.

Variables	Models
Age	Full, Anthropometric (Full, Trimmed)
Area of the normalized statokinesigram	Full, Balance
BMI	Full, Anthropometric (Full, Trimmed)
Centroid of the EMG activity	Full, Neuromuscular (Full, Centroid)
EMG entropy	Full, Neuromuscular (Full, Entropy)
Entropy of the movement	Full, Biomechanical (Full)
Foot barycentre trajectory	Full, Biomechanical (Full, Trimmed)
Ground–reaction force ratio	Full, Balance
High-density EMG	CNN
Height	Full, Anthropometric (Full)
Maximum amplitude of the movement	Full, Biomechanical (Full, Trimmed)
Maximum angle to maximum angle	Full, Biomechanical (Full)
Sex	Full, Anthropometric (Full, Trimmed)
Time to maximum amplitude	Full, Biomechanical (Full, Trimmed)
Time to maximum angle	Full, Biomechanical (Full)
Inter-subject variation	Full, Biomechanical (Full)
Intra-subject variation	Full, Biomechanical (Full)
Weight	Full, Anthropometric (Full)

**Table 4:** Convolutional neural network architecture.

Layer (Type)	Output Shape	Nb of Parameters
input_1 (InputLayer)	(None, 26, 10, 6000, 1)	0
conv3d (Conv3D)	(None, 26, 10, 6000, 8)	1448
conv3d_1 (Conv3D)	(None, 26, 10, 6000, 8)	11,528
max_pooling3d (MaxPooling3D)	(None, 13, 5, 599, 8)	0
batch_normalization (BatchNormalization)	(None, 13, 5, 599, 8)	32
conv3d_2 (Conv3D)	(None, 13, 5, 599, 16)	23,056
conv3d_3 (Conv3D)	(None, 13, 5, 599, 16)	46,096
max_pooling3d_1 (MaxPooling3D)	(None, 6, 2, 58, 16)	0
batch_normalization_1 (BatchNormalization)	(None, 6, 2, 58, 16)	64
conv3d_4 (Conv3D)	(None, 6, 2, 58, 32)	92,192
conv3d_5 (Conv3D)	(None, 6, 2, 58, 32)	184,352
max_pooling3d_2 (MaxPooling3D)	(None, 3, 1, 4, 32)	0
batch_normalization_2 (BatchNormalization)	(None, 3, 1, 4, 32)	128
flatten (Flatten)	(None, 384)	0
dense (Dense)	(None, 64)	24,640
dropout (Dropout)	(None, 64)	0
dense_1 (Dense)	(None, 64)	4160
dropout_1 (Dropout)	(None, 64)	0
dense_2 (Dense)	(None, 1)	520

**Table 5:** DNN maximum performance.

Model	Accuracy (Test)	Accuracy (Validation)	Epochs
Full model	99.88	93.30	100
Anthropometric	92.84	94.40	300
Trimmed	94.40	98.19	300
Biomechanical	83.05	84.47	90
Trimmed	88.29	88.45	398
Trimmed Normalized	87.40	92.96	194
Neuromuscular	83.27	88.07	15
Entropy	76.21	87.30	104
Centroid	85.37	90.00	50
3D Convolutional Neural Network	100.00	73.75	23
Balance	68.90	74.73	68

**Table 6:** Factor analysis results for movements at preferred speed.

Movements	Group	Age	BMI	Sex	Max. ROM	Time to Max. ROM	Traj. Entropy	Traj. Intra-Var.	Traj. Inter-Var.	EMG Centroid Y Pos.	EMG Entropy l. Low Back	EMG Entropy r. Low Back	GFR Ratio
Back ext.	0.292 ^*^	0.381 ^**^				0.958 ^‡^	−0.736 ^‡^			0.338 ^*^			
Back flex.	0.520 ^‡^		0.405 ^**^				0.553 ^‡^			0.538 ^‡^	−0.420 ^†^		
Lat. trunk flex. l.	0.368 ^*^	0.716 ^‡^	0.395 ^**^	−0.403 ^**^		0.622 ^‡^	−0.380 ^**^		0.618 ^‡^				−0.622 ^‡^
Lat. trunk flex. r.	0.308 ^*^	0.442 ^†^	0.818 ^‡^							0.387 ^**^			−0.357 ^*^
Trunk rot. left	−0.378 ^*^												0.943 ^‡^
Trunk rot. right	0.387 ^**^	0.494			0.587 ^‡^	−0.345 ^*^			0.373 ^*^	0.412 ^†^			

*: *p* < 0.05. **: *p* < 0.01. †: *p* < 0.005. ‡: *p* < 0.001.

**Table 7:** Factor analysis results for movements at maximum speed.

Movements	Group	Age	BMI	Sex	Max. ROM	Time to Max. ROM	Traj. Entropy	Traj. Intra-Var.	Traj. Inter-Var.	EMG Centroid Y Pos.	EMG Entropy l. Low Back	EMG Entropy r. Low Back	GFR Ratio
Back ext.	0.370 ^*^					0.786 ^‡^	−0.645 ^‡^			0.338 ^*^			
Back flex.	0.237				0.582 ^‡^	0.440 ^†^		−0.923 ^‡^	−0.324 ^*^				
Lat. trunk flex. l.	0.387 ^**^					0.504 ^‡^		0.299 ^*^	0.872				−0.481 ^‡^
Lat. trunk flex. r.	0.221	0.326 ^*^				0.614 ^‡^	−0.418 ^†^			0.429 ^†^		−0.585 ^‡^	
Trunk rot. left	0.531 ^‡^	0.319 ^*^			0.547 ^‡^				0.356 ^*^				−0.300 ^*^
Trunk rot. right	0.321 ^*^	0.532 ^‡^	0.349 ^*^			0.746 ^‡^	−0.594 ^‡^	−0.424 ^†^		0.405 ^†^		−0.597 ^‡^	

*: *p* < 0.05. **: *p* < 0.01. †: *p* < 0.005. ‡: *p* < 0.001.

## Data Availability

The data presented in this study are available on request from the corresponding author. The data are not publicly available due to logistical reasons.

## Abbreviations

The following abbreviations are used in this manuscript:

BMI	Body mass index
CNN	Convolutional Neural Network
DNN	Deep Neural Network
EMG	Electromyography
EMG centroid Y pos.	Y position of the total back EMG centroid
EMG entropy l. low back	Entropy of the EMG of the left lower back
EMG entropy r. low back	Entropy of the EMG of the right lower back
FA	Factor Analysis
GFR	Ground Force Reaction
HD EMG	High Density Electromyography
LBP	Low Back Pain
max. ROM	Maximum amplitude of the left shoulder trajectory
NSLBP	Non-Specific Low Back Pain
RMS	Root mean square
RMSE	Root mean square error
SVM	Support vector machine
Time to max. ROM	Time to maximum amplitude of the left shoulder trajectory
Traj. entropy	Entropy of the movement of the left shoulder trajectory
Traj. intra-var.	Intra-subject variation in the left shoulder trajectory
Traj. inter-var.	Inter-subject variation in the left shoulder trajectory
